# Fabrication of Cementitious Microfiltration Membrane and Its Catalytic Ozonation for the Removal of Small Molecule Organic Pollutants

**DOI:** 10.3390/membranes11070532

**Published:** 2021-07-14

**Authors:** Jingyi Sun, Shan Liu, Jing Kang, Zhonglin Chen, Liming Cai, Yuhao Guo, Jimin Shen, Zhe Wang

**Affiliations:** 1 State Key Laboratory of Urban Water Resource and Environment, School of Environment, Harbin Institute of Technology, Harbin 150090, China; sunjingyihit@163.com (J.S.); liumountainhit@163.com (S.L.); cailiminghit@163.com (L.C.); 1190401204@stu.hit.edu.cn (Y.G.); shenjimin@hit.edu.cn (J.S.); 2 School of Environmental Science and Safety Engineering, Tianjin University of Technology, Tianjin 300384, China; wangzh203@163.com

**Keywords:** cementitious microfiltration membrane, bending strength, alkaline buffering effect, catalyze ozonation

## Abstract

In this study, a low-cost cementitious microfiltration membrane (CM) with a catalytic ozone oxidation function for the removal of organic pollutants was fabricated by using cementitious and C-10 μm silica powders at a certain silica–cementitious particle ratio (s/c). The effect of the s/c on the pore size distribution and mechanical strength of the membrane was investigated. The membrane pore size showed a bimodal distribution, and the higher the s/c, the closer the second peak was to the accumulated average particle size of silica. The increase in the s/c led to a decrease in the bending strength of the membrane. The cross-sectional morphology by SEM and crystal structure by XRD of CMs confirmed that a calcium silicate hydrate gel was generated around the silica powder to improve the mechanical strength of the CM. Considering the bending strength and pore size distribution of CMs, s/c = 0.5 was selected as the optimal membrane fabrication condition. The FT-IR results characterizing the surface functional groups of CMs were rich in surface hydroxyl groups with the ability to catalyze ozone oxidation for organic pollutant removal. Six small molecule organic pollutants were selected as model compounds for the efficiency experiments via a CM–ozone coupling process to prove the catalytic property of the CM. The CM has an alkaline buffering effect and can stabilize the initial pH of the solution in the catalytic ozonation process. The reuse experiments of the CM–ozone coupling process demonstrated the broad spectrum of the CM catalytic performance and self-cleaning properties. The results of this study provide the basis and experimental support to expand the practical application of CMs.

## 1. Introduction

The extensive use of chemicals and pharmaceuticals in daily human activities leads to the release of a wide range of organic compounds into the surrounding environment [1]. Large molecular organic compounds can be removed by conventional processes in water treatment, such as rejection by ultrafiltration membranes and removal by a disinfection process [2], while small molecular organic pollutants cannot be effectively degraded by conventional processes in wastewater treatment plants (WWTPs) and are usually removed by advanced treatments, such as catalytic ozonation [3], catalytic peroxydisulfate [4], and photochemical processes [5]. In these advanced oxidation processes, the catalysts are difficult to separate and recover [6].

A membrane is a type of material with selective and separation functions, which separates, purifies, and concentrates different components, in a purely physical process with no phase change, saving energy, and with a simple operation [7,8]. Membrane technology is considered to be key for the currently developing third generation of urban drinking water purification processes [9]. Microfiltration membranes, as the basic membrane separation technology, have the advantages of saving energy and being driven by low pressure [10]. However, for small molecule organic contaminants, microfiltration membranes are unable to remove them directly by retention due to their large pore sizes. Therefore, the development of functional microfiltration membranes is optimal to expand their application. The membrane-catalyzed ozonation process loads the catalyst onto the membrane surface or inside the membrane pores [11]; this technique has good recovery properties and long-term operation in water treatments.

Cementitious materials are widely used due to their low-cost raw materials and can easily be shaped by hydration [12,13]. Dong et al. [14,15] prepared a novel silicate cement support by freeze casting, using TBA/H_2_O as a template, a water flux of 406.07–1514.69 L m^−2^ h^−1^, and a membrane flexural strength of 1.12–12.48 MPa. In our previous study [16,17], a cementitious microfiltration membrane was fabricated using quartz and cement as raw materials by dry press molding, and the membrane could catalyze the ozone oxidation of *p*-chloronitrobenzene (*p*-CNB), which increased the *p*-CNB removal rate by 50% compared to that of sole ozonation. However, prior studies could not guarantee both a high water flux and a high mechanical strength of the membrane, which greatly limits the practical application of cementitious membranes.

In this study, based on the previous fabrication method of an aluminosilicate-based microfiltration membrane [18], a cementitious microfiltration membrane (CM) was prepared with cementitious and amorphous silica powders (C-10 μm) as raw materials. Effects of the silica–cementitious ratio (s/c) on the pore size distribution, mechanical strength, pure water flux (PWF), and porosity of the membrane were investigated to obtain the optimal membrane conditions. Benzophenone-4 (BP-4) is one of the most widely used UV filters [3]; it is added to sunscreen, lotion, lipstick, and other personal care products (PCPs) [19] and is frequently detected in aquatic environments [20,21,22]. Furthermore, nitrobenzene [23], *p*-chlorophenol (*p*-CP) [24], *p*-chloronitrobenzene (*p*-CNB) [25], and p-chlorobenzoic acid (*p*-CBA) [26] are common organic pollutants as pesticides, dye intermediates, etc., widely used in human activities. The membrane-catalyzed ozonation efficiency and reaction kinetics of the above-mentioned model pollutants were studied. A leveling effect of the membrane-catalyzed ozonation system of the initial pH in solution was also found. The membrane reuse experiment provides guidance for the practical applications of CMs.

## 2. Materials and Methods

### 2.1. Materials and Reagents

The main raw materials for the membrane fabrication were cementitious powder (PO 42.5, Tian E^®^, Harbin, China) and silica. Silicon dioxide powder was purchased from Macklin^®^ Shanghai, China, named C-10 μm. Nitrobenzene (99% purity), *p*-CNB (99% purity), *p*-CA (98% purity), *p*-CP (99% purity), and *p*-CBA (99% purity) were all obtained from Sigma-Aldrich (Sigma-Aldrich Inc., St. Louis, MI, USA), and BP-4 (98% purity) was obtained from J&K (Beijing, China), with a stock solution concentration of 100 mg/L. Bovine serum albumin (BSA, MW = 66.5 kDa) was purchased from Aobox (Beijng, China). Ultrapure water was supplied by a Milli-Q^®^ system (resistivity > 18.0 M cm) and used in all experiments.

### 2.2. Experimental Methods

#### 2.2.1. Membrane Fabrication Process

In this study, cementitious microfiltration membranes were fabricated by a dry press molding method using cementitious powder and C-10 μm as raw materials. The two raw materials were mixed in a certain s/c ratio as dry materials by premixing at 30 r/s for 2 min. Ultrapure water was added to the dry materials at a water-to-cementitious powder ratio (w/c) of 0.2 and mixed rapidly at a speed of 100 r/s for 1 min. The obtained slurry was transferred into a mold with an inner diameter of 50 mm and thickness of 5 mm and subjected to a pressure of 6 MPa for 1 min to obtain the pressed paste. Both the pressed paste and mold were cured in the standard curing box (Shanghai Bluepard Instruments Co. Ltd., Shanghai, China) at a temperature of 20 °C and relative humidity (RH) of 90% for 24 h. Then, the membrane was demolded and continued to be cured for 13 d. CMs with different s/c ratios were obtained using the same fabrication method.

#### 2.2.2. CM–Ozone Coupling Experiments

CM–ozone coupling experiments for the removal of organic pollutions were performed with 0.064 mM, and 250 mL of an individual organic pollutant as influent under consistent ozone conditions in batch, and the process of the experiments is shown in Figure S1. The influent was continuously circulated through the CMs in a closed vessel and sampled at certain times to determine the concentration of organic pollutions. Ozone was continuously generated by ionized pure oxygen through an ozone generator (COM-AD-01 Anseros, Tübingen-Hirschau, Germany). The ozone concentration in the solution was 0.5 mg/L, which was determined via the indigo method [27]. All experiments were repeated in triplicate. Membrane reuse experiments were performed by repeating the above experiments with different organic batches for six times, using the same membrane for each experiment. Additionally, there was no backwash between each cycle. The concentration of pollutants in each cycle was measured, and the reaction kinetics of membrane catalytic ozone oxidation to remove organic matter was calculated.

### 2.3. Characterization of CMs and Analytical Methods

The pore size distribution of membranes was measured by a pore size analyzer (PSDA, Nan Jing Gao Qian Functional Materials Co., Ltd., Nanjing, China), based on the “Standard Test Methods for Pore Size Characteristics of Membrane Filters by Bubble Point and Mean Flow Pore Test” ASTM F316 standard [28]. The surface tension of the wetting liquid was 16 mN m^−1^. PWF of the membrane was measured by weighing the mass of water permeated by the membrane in unit time. The effective filtration area of the membrane is 7.07 cm^2^. The analysis balance was purchased from Mettler Toledo (MS105DU, Greifensee, Switzerland), and the minimum display of the analytical balance is 0.01 mg. The measured method and schematic of PWF are shown in Text S2 and Figure S2. The particle size distribution of the raw material for membranes was assessed using a laser particle size analyzer, the Mastersizer 2000 instrument (Malvern Instrument Co., Ltd., Malvern, UK). The rejection efficiency of BSA by the CM was tested to demonstrate the separation performance of the CM. The testing method is shown in Text S1. The microstructure of raw materials and the cross-section of the membranes were observed by field-emission scanning electron microscopy (SEM, Sigma 500 Zeiss Corp., Jena, Germany). The mechanical strength of the membrane was characterized by three-point bending strength and tested via a universal strength testing machine (Instron 5569, Instron Corp., Norfolk County, MA, USA). The phase compositions and crystalline structures of the membranes were determined by X-ray diffraction (XRD, D8 ADVANCE, Bruker Co, Bearrica, Germany). The surface functional groups of the membranes were tested by Fourier-transform infrared spectroscopy (FT-IR, Spectrum One, PerkinElmer, Waltham, MA, USA). The pH value of the solution was determined via a pHS-3C meter (Beijing Kewei Yongxing Instrument CO., LTD., Beijing, China).

All of the organic models were determined by ultra-performance liquid chromatography (UPLC, Agilent 1290 Infinity II, Santa Clara, CA, USA) and separated on an Agilent ZORBAX SB-C18 (4.6 × 150 mm, 5 μm) column. The column temperature was 30 °C, and the injection volume was 10 μL. The mobile phases of nitrobenzene, *p*-CNB, and *p*-CA were all methanol/water ratios of 70:30 (*v*/*v*). The detection wavelengths of the UV detector were 262, 260, and 244 nm, respectively. The mobile phase of *p*-CP was acetonitrile and water with a 50:50 (*v*/*v*) ratio, and mobile phases of *p*-CBA and BP-4 were 0.1% formic acid in acetonitrile/water ratios of 60:40 and 40:60 (*v*/*v*), respectively. The detection wavelengths of the UV detector were 220, 236, and 285 nm, respectively. The isocratic elution of organic compounds was performed at a flow rate of 1.0 mL min^−1^, except for *p*-CBA, which was performed at 0.8 mL min^−1^.

## 3. Results and Discussion

### 3.1. Fabrication and Characterization of CMs

#### 3.1.1. Selection of s/c in the Membrane Fabrication Process

**Figure 1:**
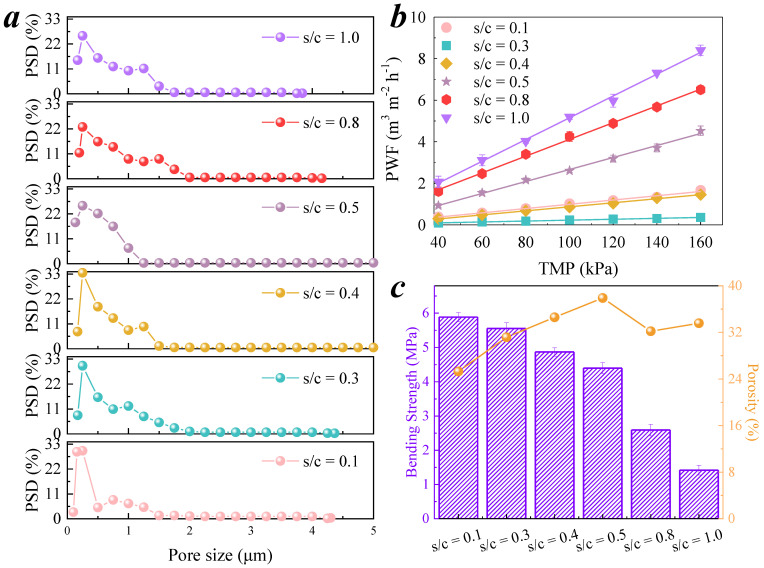
Characterization of CMs fabricated by a series of s/c ratios. (**a**) Pore size distribution of CMs; (**b**) PWF of CMs; (**c**) bending strength and porosity of CMs.

The raw materials for membrane fabrication were C-10 μm, with a volume average particle diameter (D[4, 3]) of 9.76 μm, and cementitious powder. The boundary particle size of C-10 μm was (D10, D90) = (2.00, 17.18), and the cementitious powder was sieved between 325 and 400 mesh, with (D10, D90) = (4.47, 46.29) and a (D[4, 3]) of 24.25 μm; the particle size distribution of raw materials is shown in Figure S3. The cementitious material was mixed with C-10 μm in s/c ratios of 0.1, 0.3, 0.4, 0.5, 0.8, and 1.0. A batch of CMs with different s/c values was obtained. The pore size distribution, PWF, bending strength, and porosity of the six membranes were measured, as shown in Figure 1.

The measured membrane pore size distribution values are shown in Table 1. The maximum pore sizes of the CMs ranged from 3.8 to 6.5 μm, as shown in Figure 1a. The average pore sizes of the CMs displayed an increasing trend with increasing s/c ratios and then suddenly decreased at s/c = 1.0. In addition, the membrane pore size could not be measured precisely to the nanometer level due to the limitations of the test machine; therefore, the mean pore size did not considerably differ, with values of approximately 0.20 μm. The membrane pore size distribution showed a bimodal distribution. The first peak was the mean pore size, which is the largest percentage of pore sizes in the membrane. The second peak increased first and then disappeared when s/c = 0.5. Subsequently, s/c continued to increase, and the second peak had a tendency to decrease, but it was still larger than the low s/c values.

The PWF values of the CMs at different TMPs are shown in Figure 1b. The change trend of PWF was in contrast to that of the average pore size, showing a trend of decreasing first and then increasing. The porosity values of the CMs are shown in Table 2, presenting a similar trend to the second peak of the pore size distribution (Figure 1c); the maximum value was 37.90% at s/c = 0.5.

Some studies have proposed that the relationship between raw materials and the pore size of membranes is in accordance with Equation (1) [17,29]:

(1) $$\frac{d_{\mathrm{mod}}}{P_{50}}\;=\;-0.515\ln\left(1-\varepsilon \right)$$

where *d*_mod_ is the modal pore diameter of the membrane, *P*_50_ is the median value of the particle size distribution of the raw materials, and *ε* is the porosity of the membrane. As it is shown in Table 2, the average porosity of the six CMs was 32.4%. Substituting this average porosity and the *d*_50_ of C-10 μm silica into Equation (1), the calculated *d*_mod_ result was 1.83 μm. Compared with the second peak in Table 1, the larger the s/c, the closer the second peak pore size is to *d*_mod_. Specifically, the larger the s/c, the closer the second peak is to the stacked average particle size of silica, while both the second peak pore size and the mean pore size are smaller than the *d*_mod_ value. This may be due to the hydration of cementitious bonds in the silica, and the hydride crystals which cut the pores formed by the stacked silica particles, thus resulting in a reduction in the pore size.

The mechanical strength of the CMs decreased from 5.89 to 1.42 MPa with increasing s/c, as shown in Figure 1c and Table 2. During the use of CMs, it was found that the CMs were very loose at s/c = 0.8 and 1.0, and their strength was too low to be reused.

**Figure 2:**
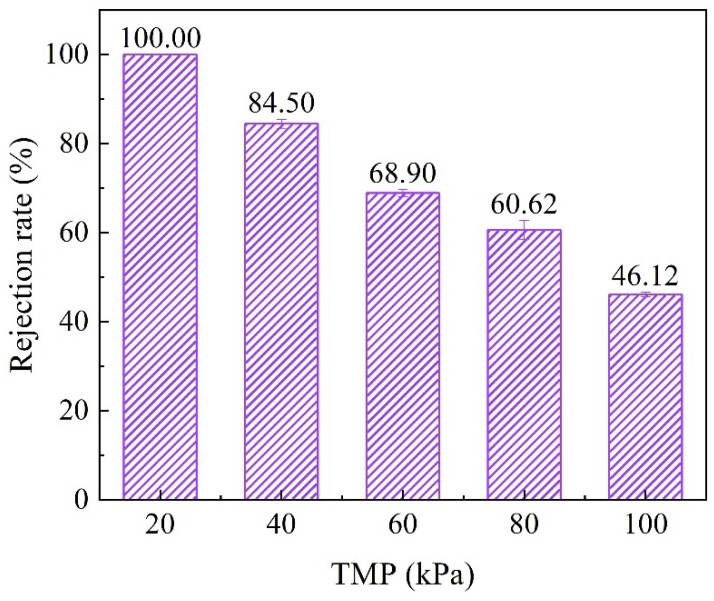
The rejection efficiency of BSA by the membrane. Conditions: [BSA]_0_ = 100 mg L^−1^. Membrane fabrication: s/c = 0.5, 20 °C, 90% RH, curing for 14 d.

When s/c = 0.5, the membrane pore size distribution was the most uniform, the porosity was the largest, and the PWF and the mechanical strength of the membrane also showed a strong performance. In summary, s/c = 0.5 was selected as the optimal ratio for the preparation of CMs. The retention of BSA by the membrane fabricated by s/c = 0.5 at a series of trans-membrane pressures (TMP) was tested, as shown in Figure 2. The rejection rate of BSA was decreased with increasing TMP, and the retention of BSA was excellent at a low TMP. A microfiltration membrane is a low-driving pressure membrane. The PWF of the membrane fabricated at s/c = 0.5 was 2617 L m^−2^ h^−1^ bar^−1^, and it was large enough to filtrate at a low TMP. This result ensures the retention rate of large molecules by the CM.

#### 3.1.2. Characterization of CMs with Series of s/c Ratios

**Figure 3:**
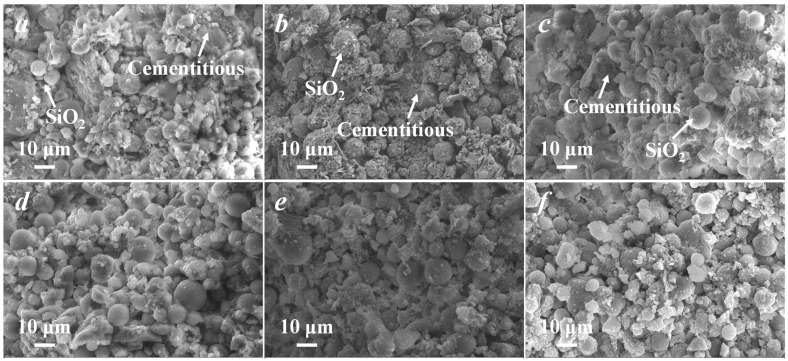
Cross-sectional morphologies of CMs fabricated by a series of s/c ratios. (**a**) s/c = 0.1; (**b**) s/c = 0.3; (**c**) s/c = 0.4; (**d**) s/c = 0.5; (**e**) s/c = 0.8; (**f**) s/c = 1.0.

The cross-sectional morphologies of CMs fabricated with different s/c ratios could be observed by SEM. The cementitious powder was irregularly blocky, as shown in Figure S4a, and the apparent morphology of C-10 μm is shown in Figure S4b, which was regular and spherical. As it is shown in Figure 3a–f, the aggregation of C-10 μm in the cross-section of the membrane increased significantly with the increase in s/c. Calcium silicate hydrate (C-S-H) is the main hydration product of the cementitious materials, and it contributed to the strength in cementitious materials [30]. The C-S-H gel generated around C-10 μm reduced, and C-10 μm was more dispersed. Silica has pozzolanic activity. This means the active silica can react with the Ca(OH)_2_ in the cementitious materials to produce C-S-H [31], which can enhance the hydration reaction, generate more hydration products, induce the cement to generate a dense microstructure, and enhance the mechanical strength of the membrane. As it is shown in Figure 3a–d, a large amount of C-S-H gel was generated around the silica powder, which firmly held the silica powder [31,32]. As it is shown in Figure 3e,f, flakes and hexagonal forms of Ca(OH)_2_ were observed. Ca(OH)_2_ contributes little to the strength of the cementitious powder, which has weak interlayer linkages and is less stable. This is most often the source of crack generation in cementitious materials [31]. In addition, the CMs were mainly bonded by cementitious hydration linkages at low s/c ratios. As the C-10 μm content increased, the contact of cementitious particles became poor and was blocked by the large amount of C-10 μm, resulting in an incomplete hydration effect, which caused the mechanical strength to decrease, the porosity to increase, and the average pore size to also increase. When the content of C-10 μm continued to increase, the high pressure in the process of membrane preparation squeezed the C-10 μm tightly together. The accumulation of particles in the membrane is mainly the extrusion accumulation of C-10 μm. The particle size of the cementitious powder was larger than that of C-10 μm, and the calculated compacted C-10 μm pore was smaller than the compacted cement pore, as in Equation (1), leading to a decrease in porosity and the average pore size of CMs at a high s/c.

**Figure 4:**
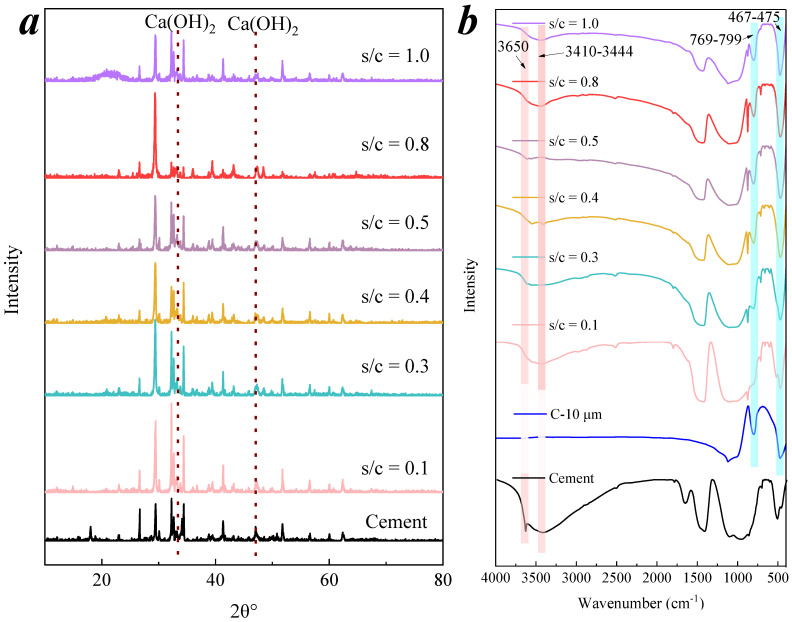
Effect of s/c on microstructure and surface functional groups of CMs. (**a**) XRD patterns of CMs with different s/c ratios; (**b**) FT-IR spectra of CMs with different s/c ratios.

The XRD spectra of CM raw materials contained multiple chemical constituents, including calcium silicate, larnite, calcium carbonate, calcium hydroxide, and quartz, as the main composition of the cementitious powder, as shown in Figure 4a. The XRD results show that the peaks of Ca(OH)_2_ in CMs were all observed. This result was also embodied in the FT-IR spectra, as shown in Figure 4b. The peak at 3650 cm^−1^ belonged to the O–H bond of Ca(OH)_2_ [33]. The peaks between 769 and 799 cm^−1^ and 467 and 475 cm^−1^ are attributed to the Si–O bonds of the raw SiO_2_ powder. The active Si–OH can react with free calcium hydroxide to enhance the hydration of the cementitious material [34,35]. The peak at 3410–3444 cm^−1^ belonged to the stretching vibration and bending vibration of Si–OH or O–H of water molecules, which may be caused by surface OH or adsorbed water [36]. Hydroxyl groups on the surface of materials may be able to catalyze ozone to produce hydroxyl radicals [16].

### 3.2. Degradation of Organic Pollutants by CM-Catalyzed Ozone

**Figure 5:**
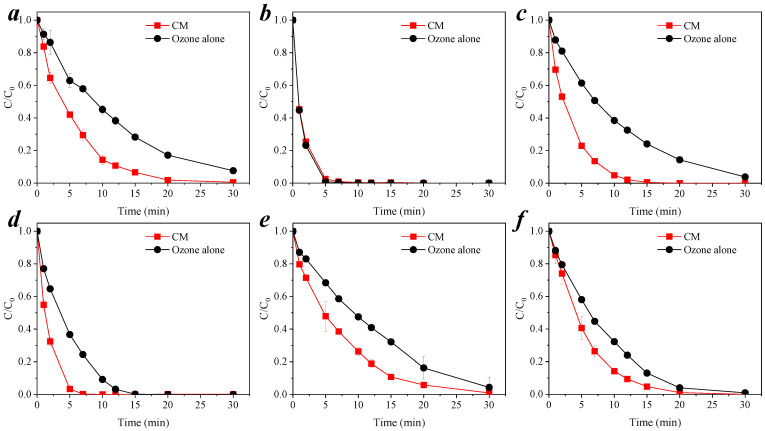
Degradation of organic compounds by CM catalytic ozonation. (**a**) Nitrobenzene; (**b**) *p*-CA; (**c**) BP-4; (**d**) *p*-CP; (**e**) *p*-CNB; (**f**) *p*-CBA. Conditions: pH = 6.9 ± 0.1, [O_3_] = 0.5 mg L^−1^, [nitrobenzene]_0_ = [*p*-CA]_0_ = [BP-4]_0_ = [*p*-CP]_0_ = [*p*-CNB]_0_ = [*p*-CBA]_0_ = 0.064 mM.

The catalytic ability of silicate microfiltration membranes to catalyze the oxidation of *p*-CNB by ozone was proposed in a previous report [16]. To investigate the broad spectrum of organic pollutant ozonation catalyzed by CM (s/c = 0.5), six small molecule organic pollutants with different functional groups, log*K*_ow_ and p*K*_a_, were chosen as model compounds which were degraded in the CM–ozone coupling process, and the results obtained are shown in Figure 5. The basic information of the six model compounds, such as chemical structure formula, p*K*_a_ and log*K*_ow_, are listed in Table S1. Except for *p*-CA, the removal of the other five model compounds by the CM–ozone coupling processes was significantly better than that of ozone alone. In order to avoid the adsorption or retention effect of the six organic compounds by the CM, the membrane adsorption experiments of the six substances by the CM were conducted without ozonation. The results show that the membrane itself had no adsorption or retention effect on the organic pollutants, as shown in Figure S5. It was demonstrated that the removal of organic matter by the CM–ozone coupling process was through catalytic oxidation.

As it is shown in Figure S6, these reactions were fitted to the pseudo-first-order kinetic, and the reaction rate constants *k*_obs_ were calculated, as shown in Table 3. For the six compounds, nitrobenzene, *p*-CA, BP-4, *p*-CP, *p*-CNB, and *p*-CBA, the *k*_obs_ of the CM–ozone coupling process were 2.13, 0.77, 2.96, 2.57, 1.69, and 1.59 times greater than those of ozone alone, respectively. *p*-CA was easily degraded by ozone [37,38]. It has been reported that the other five compounds, nitrobenzene [23], BP-4 [3], *p*-CP [24], *p*-CNB [11], and *p*-CBA [26], are more difficult to be removed by ozone molecules alone and require catalytic ozone to generate more oxidizing radicals for degradation. These results confirm that CMs can catalyze ozone to generate free radicals [16] to degrade organic pollutants, which were not easily oxidized by ozone molecules.

### 3.3. Effect of Initial pH

It has been reported in many studies that the pH has a significant effect on the reaction rate of oxidation and catalytic oxidation systems for the removal of organic compounds [39]. Under alkaline conditions, OH^−^ reacts with ozone molecules in solution to form the more oxidizing OH, thus accelerating the removal efficiency of pollutants [23,39].

**Figure 6:**
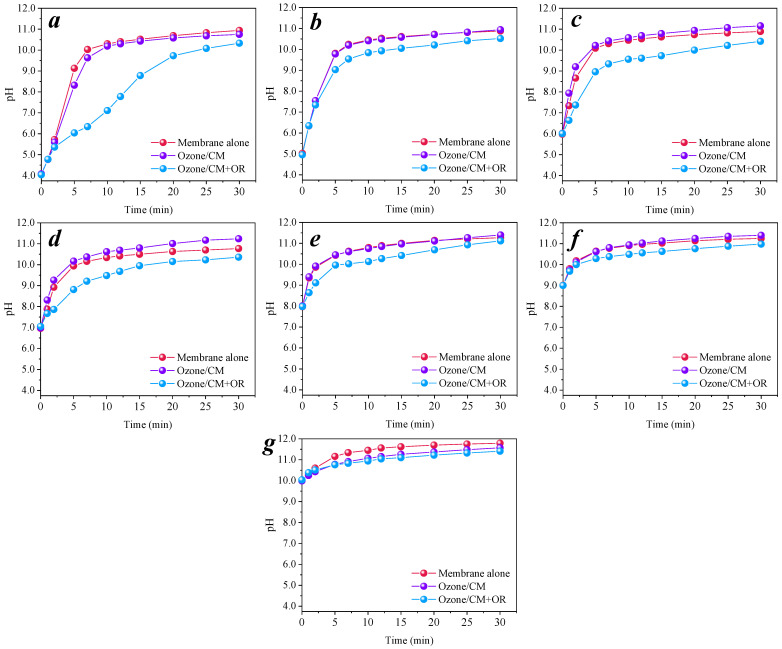
Change trends of pH in solution with different initial pH values. (**a**) pH_initial_ = 4.0; (**b**) pH_initial_ = 5.0; (**c**) pH_initial_ = 6.0; (**d**) pH_initial_ = 7.0; (**e**) pH_initial_ = 8.0; (**f**) pH_initial_ = 9.0; (**g**) pH_initial_ = 10.0. Conditions: [O_3_] = 0.5 mg L^−1^, [nitrobenzene]_0_ = 0.064 mM.

It was found that the pH of pure water increased after the membrane was introduced. For this phenomenon, the pH change trends of the solution were monitored in three processes: placing the membrane alone in pure water, which was named the membrane alone system; continuously pumping ozone into the pure water with membranes, which was named the Ozone/CM system; and adding the model pollutant nitrobenzene to the membranes with ozone coexisting in the pure water, which was named the Ozone/CM + OR system. The changes in pH in the three process solutions were measured at initial pH = 4.0, 5.0, 6.0, 7.0, 8.0, 9.0, and 10.0, as shown in Figure 6. The pH of all three systems increased with time. The pH trend of the Ozone/CM + OR system was slower than that of the system without nitrobenzene, and the pH trend of the Ozone/CM system was slower than that of the membrane alone system. This was due to the fact that the system with ozone and nitrobenzene will consume a certain amount of OH^−^ in the solution to produce free radicals; nitrobenzene, especially, will react directly with the free radicals, which will accelerate the consumption of OH^−^ and lead to a slower pH rise. When the nitrobenzene was completely degraded, the pH of the three systems tended to equilibrium. As it is shown in Figure 6, the initial pH of the solution had little effect on the 30 min endpoint pH of the three systems, and the final pH was in the range of 10.33–11.40, which is alkaline. It has been reported in the literature that a cement-like base material is an alkaline buffer which can stabilize the solution pH at about 10.5 after soaking for 28 days [40]. This result was confirmed by this study.

**Figure 7:**
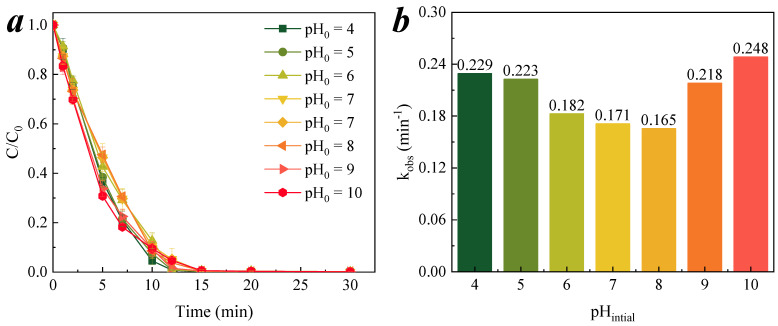
Effect of initial pH on nitrobenzene degradation and reaction kinetics of CM catalytic ozonation. (**a**) Degradation of nitrobenzene in different initial pH conditions; (**b**) the corresponding rate constants *k*_obs_ for the catalytic ozonation of nitrobenzene at different initial pH. Conditions: [O_3_] = 0.5 mg/L, [nitrobenzene]_0_ = 0.064 mM.

The removal efficiency of nitrobenzene at various initial pH values was determined, as shown in Figure 7a; the results were fitted to the pseudo-first-order reaction kinetics, and the reaction rate constants are shown in Figure 7b. These seven *k*_obs_ values were statistically calculated, where the standard deviation was 0.032, and the coefficient of variation was 0.14; the smaller these two values are, the less measures of dispersion are, and these values are closer to the average. This indicated that the alkaline buffering effect of the cementitious membrane was strong, and that the initial pH has less of an effect on the removal efficiency for nitrobenzene.

### 3.4. Reusability of CMs

**Figure 8:**
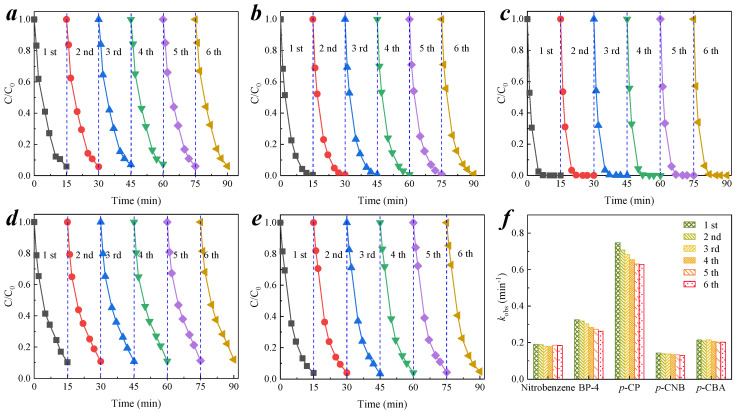
Reuse of the CM–ozone catalyst process in the degradation of organic pollutants. Degradation of (**a**) nitrobenzene; (**b**) BP-4; (**c**) *p*-CP; (**d**) *p*-CNB; and (**e**) *p*-CBA by CM-catalyzed ozonation; (**f**) reaction kinetics of the CM-catalyzed ozonation process. Conditions: pH = 6.9 ± 0.1, [O_3_] = 0.5 mg L^−1^, [nitrobenzene]_0_ = [BP-4]_0_ = [*p*-CP]_0_ = [*p*-CNB]_0_ = [*p*-CBA]_0_ = 0.064 mM.

In practice, the stability of membranes is important for reuse. Based on the results of Section 3.2, five organic pollutants with better catalytic removal efficiency in the CM-catalyzed ozone oxidation system were selected, which were nitrobenzene, BP-4, *p*-CP, *p*-CNB, and *p*-CBA. As it is shown in Figure 8a–e, the removal efficiency of five organic pollutants, by the CM–ozone coupling process, was stable. After six reuse cycles, the removal efficiency of BP-4 by the CM–ozone process did not decrease significantly. The pseudo-first-order kinetics of the reaction were fitted, as shown in Figure 8f, to calculate the *k*_obs_, as shown in Table S2. The sixth reaction *k*_obs_ of nitrobenzene, BP-4, *p*-CP, *p*-CNB, and *p*-CBA were decreased by 3.33%, 19.17%, 15.84%, 9.14%, and 5.17% compared with the first reaction, respectively. Only the *k*_obs_ of BP-4 and the *k*_obs_ of *p*-CP were decreased by more than 10%, while the *k*_obs_ of these two substances for the catalytic oxidation were already large enough. This proved that the CM-catalyzed ozonation for pollutant removal was stable and reusable for a wide range of organic compounds. CM–ozone coupling can catalyze ozone to form reactive oxygen radicals, and these strong oxidizing radicals can effectively decompose parent organic pollutants into smaller molecule oxidation products, which can smoothly pass through the micron-level membrane pores and inhibit the formation of membrane fouling on the membrane surface. The self-cleaning property of the membrane was realized [41,42]. The stability of the reaction rate constants for the five targets in this experiment also proved that multiple uses did not have a significant effect on the target degradation. This has significance, promoting the practical applications of CMs.

## 4. Conclusions

In this study, a low-cost, sintering-free microfiltration membrane was prepared by mixing cementitious silica and C-10 μm at a certain s/c ratio. The membrane pore sizes exhibited a bimodal distribution. The higher the s/c, the closer the second pore size peak was to the C-10 μm stacking pore size. An increase in s/c led to a decrease in the membrane’s mechanical strength. Combining membrane porosity and PWF, s/c = 0.5 was chosen as the optimal membrane ratio. The CM has a good retention efficiency on BSA at a low TMP. A variety of small molecule organic pollutants can be removed by CM catalytic ozone oxidation with a significantly improved reaction rate constant compared to that of ozone molecule oxidation alone. The performance of membrane-catalyzed ozone was demonstrated to be broad spectrum. In the experiments of the effect of the pH on the membrane-catalyzed ozonation of organic pollutants, it was found that CMs had an alkaline buffering effect, and the initial pH of the solution had no significant effect on nitrobenzene removal. In the CM–ozone coupling reuse experiment, the reaction rate constants for the removal of varieties of organic compounds by six consecutive reuse cycles did not change obviously, indicating the stability of the catalytic performance of the CM and its self-cleaning properties. The results of this study provide support for the practical applications of CMs.

## Figures and Tables

**Table 1:** Pore size distribution of membranes fabricated by a series of s/c ratios.

s/c	Largest Pore Size μm	Average Pore Size μm	Mean Pore Size μm	Second Peak Pore Size μm
0.1	4.301	0.336	0.25	0.75
0.3	4.366	0.358	0.20	1.00
0.4	5.706	0.554	0.15	1.25
0.5	6.584	0.633	0.25	-
0.8	4.156	0.724	0.25	1.5
1	3.839	0.618	0.25	1.25

**Table 2:** Effect of s/c on membrane properties: porosity, bending strength, and PWF.

s/c	Porosity	Bending Strength	PWF
	%	MPa	L m^−2^ h^−1^ bar^−1^
0.1	25.31	5.89	984
0.3	31.15	5.55	228
0.4	34.58	4.87	847
0.5	37.90	4.40	2617
0.8	32.18	2.59	4117
1.0	33.56	1.42	5147

**Table 3:** Reaction kinetics of organic pollutant removal by CM catalytic oxidation.

Organic Compounds	CM		Ozone Alone	
	*k*_obs_ (min^−1^)	R^2^	*k*_obs_ (min^−1^)	R^2^
Nitrobenzene	0.1837	0.996	0.0861	0.995
*p*-CA	0.7331	0.998	0.9521	0.991
BP-4	0.2849	0.998	0.0964	0.999
*p*-CP	0.6836	0.995	0.2664	0.963
4-CNB	0.1406	0.996	0.0834	0.978
*p*-CBA	0.2039	0.998	0.1286	0.986

Conditions: pH = 6.9 ± 0.1, [O_3_] = 0.5 mg L^−1^, [nitrobenzene]_0_ = [*p*-CA]_0_ = [BP-4]_0_ = [*p*-CP]_0_ = [*p*-CNB]_0_ = [*p*-CBA]_0_ = 0.064 mM.

## Data Availability

The data presented in this study are available on request from the corresponding author.

## Supplementary Materials

The following are available online at https://www.mdpi.com/article/10.3390/membranes11070532/s1, Text S1. Testing method of BSA rejection efficiency by CM. Text S2. Measured method of PWF of the membrane. Table S1. Basic information of selected organic compounds. Table S2. Reaction kinetics of organic pollutant removal by CM–ozone catalyst process reuse experiments. Figure S1. Schematic diagram of the CM–ozone coupling process to remove organic pollutants. Figure S2. Schematic for PWF of the membrane measured. Figure S3. Particle size distribution of raw materials of the fabricated cementitious membranes. Figure S4. Apparent morphology of raw materials for membrane fabrication. Figure S5. Adsorption of the six pollutants by the CM. Figure S6. Reaction kinetics of organic compound ozonation catalyzed by CMs.
