# Supplementary material for: Fabrication of Cementitious Microfiltration Membrane and Its Catalytic Ozonation for the Removal of Small Molecule Organic Pollutants
Source: Membranes (Basel). 2021 Jul 14;11(7):532. doi: 10.3390/membranes11070532 (PMC8307055; doi:10.3390/membranes11070532)
Supplement: Supplementary file 1 [file membranes-11-00532-s001.zip › membranes-1285210-supplementary.pdf]

Supplementary materials

Fabrication of Cementitious Microfiltration Membrane and Its Catalytic Ozonation for the Removal of Small Molecule Organic Pollutants

Jingyi Sun [1], Shan Liu [1], Jing Kang [1,*], Zhonglin Chen [1,*], Liming Cai [1], Yuhao Guo [1], Jimin Shen [1] and Zhe Wang [2]

[1] State Key Laboratory of Urban Water Resource and Environment, School of Environment, Harbin Institute of Technology, Harbin 150090, China; sunjingyihit@163.com (J.S.); liumountainhit@163.com (S.L.); cailiminghit@163.com (L.C.); 1190401204@stu.hit.edu.cn (Y.G.); shenjimin@hit.edu.cn (J.S.)

[2] School of Environmental Science and Safety Engineering, Tianjin University of Technology, Tianjin 300384, China; wangzh203@163.com

* Correspondence: jingkanghit@163.com (J.K.); zhonglinchen@hit.edu.cn (Z.C.); Tel: +86-0451-86283001 (J.K.); +86-0451-86287000 (Z.C.)

Content in this SI files

Text S1 Testing method of BSA rejection efficiency by CM.

Text S2 Measured method of PWF of the membrane.

Table S1. Basic information of selected organic compounds.

Table S2 Reaction kinetics of organic pollutants removal by CM–ozone catalyst process reuse experiments.

Fig. S1 Schematic diagram of the CM-ozone coupling process to removal organic pollutant.

Fig. S2 Schematic for PWF of the membrane measured.

Fig. S3 Particle size distribution of raw materials of the fabricated cementitious membranes fabricated.

Fig. S4 Apparent morphology of raw materials for membrane fabrication.

Fig. S5 Adsorption of the six pollutants by the CM.

Fig S6 Reaction kinetics of organic compound ozonation catalyzed by CMs.

Text S1 Testing method of BSA rejection efficiency by CM.

The separation performance of CCMs was demonstrated by the rejected BSA based on the Bradford method [1, 2]. The BSA rejection (R_{BSA}) experiments were performed in a dead-end filtration cell in a laboratory-scale made setup. The weight of feed solution filtering the membrane in unit time under a series of pressures (20–100 kPa) is investigated, according to following **Equation. (1):**

$$R_{BSA} = \left(1 - \frac{C_P}{C_0}\right) \times 100\% \tag{1}$$

Where R_{BSA} is the rejection rate of BSA (%), C_P is the concentration of BSA in permeate water, and C_0 is the concentration of BSA in feed water. The concentration of 100 mg L^{-1} BSA solution was used as feed solution.

Text S2 Measured method of PWF of the membrane.

By controlling the filter pressure, the mass of pure water passed through the membrane within a certain period of time can be used to calculate the pure water flux of the membrane according to **Equation. S1**:

$$J_{W}=\frac{10\times m_{W}}{A\times\rho\times t} \quad (1)$$

where J_W is the pure water flux of the membrane (L m^{-2} h^{-1}), m_W is the mass of the permeate pure water (g), A is the effective filtration area (cm^2), which value is 7.068 cm^2, ρ is the density of water at 25 °C, which value is 0.99705 g cm^{-3}, and t is the penetration time (h). The amount of deionized water that can be filtered by the membrane under a series trans-membrane pressure (40–160 kPa) was investigated. All experiments were repeated three times.

Table S1. Basic information of selected organic compounds.

Name	Structural formula	Chemical formula	Molecular weight	pK_a	logK_{ow}
Nitrobenzene	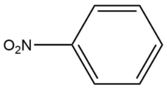	$C_6H_5NO_2$	123.111	3.98	1.85
p-chloroaniline	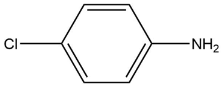	C_6H_6ClN	127.57	3.982	1.83
Benzophenone-4	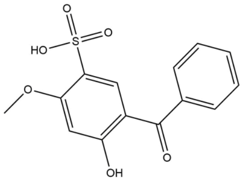	$C_{14}H_{12}O_6S$	308.31	5.993	1.89
p-chlorophenol	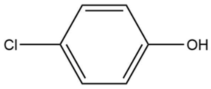	C_6H_5ClO	128.55	9.41	2.39
p-chloronitrobenzene	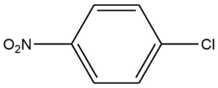	$C_6H_4ClNO_2$	157.553	-	2.39
p-chlorobenzoic acid	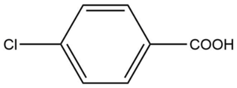	$C_7H_5ClO_2$	156.565	3.98	2.65

Table S2. Reaction kinetics of organic pollutants removal by CM–ozone catalyst process reuse experiments.

Reuse time	Nitrobenzene		BP-4		*p*-CP		*p*-CNB		*p*-CBA	
	k_{obs} min^{-1}	R^2	k_{obs} min^{-1}	R^2	k_{obs} min^{-1}	R^2	k_{obs} min^{-1}	R^2	k_{obs} min^{-1}	R^2
1 st	0.191	0.993	0.325	0.993	0.747	0.993	0.143	0.993	0.214	0.998
2 nd	0.189	0.996	0.318	0.991	0.688	0.996	0.138	0.992	0.209	0.996
3 rd	0.180	0.997	0.303	0.995	0.683	0.995	0.137	0.990	0.216	0.989
4 th	0.177	0.995	0.283	0.998	0.634	0.997	0.134	0.986	0.207	0.993
5 th	0.185	0.995	0.273	0.997	0.571	1.000	0.133	0.986	0.203	0.992
6 th	0.184	0.995	0.262	0.999	0.559	1.000	0.130	0.988	0.203	0.998

Conditions: pH = 6.9±0.1, $[O_3]$ = 0.5 mg L^{-1}, $[\text{nitrobenzene}]_0$ = $[\text{BP-4}]_0$ = $[p\text{-CP}]_0$ = $[p\text{-CNB}]_0$ = $[p\text{-CBA}]_0$ = 0.064 mM.

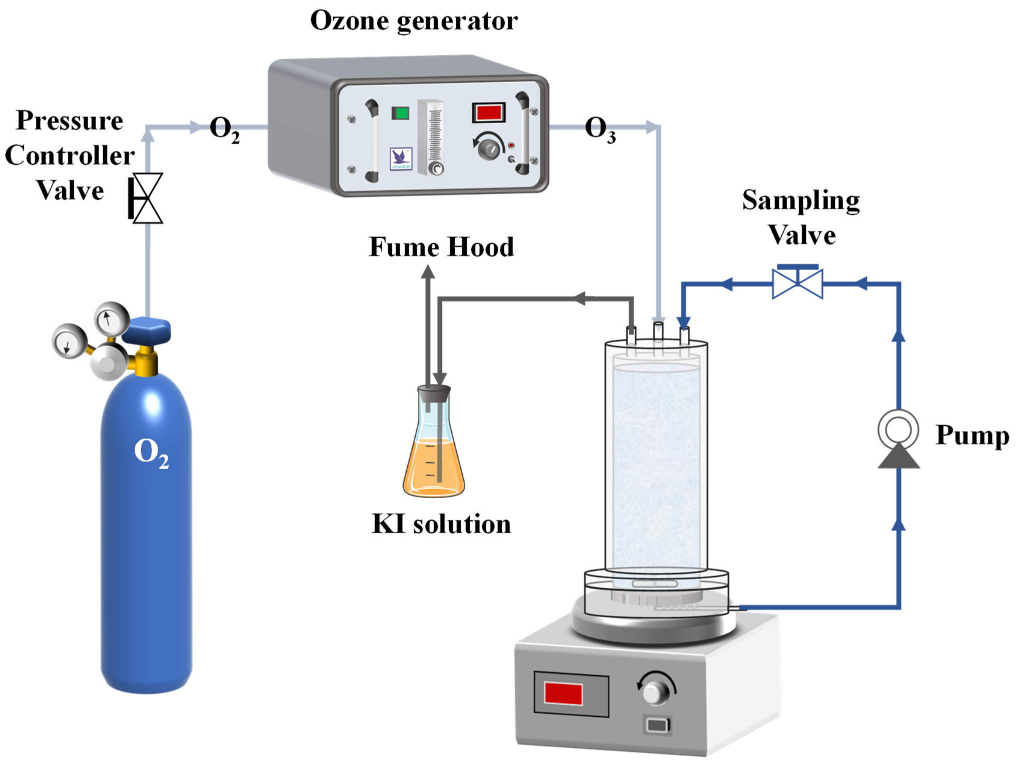

Figure S1. Schematic diagram of the CM-ozone coupling process to removal organic pollutant.

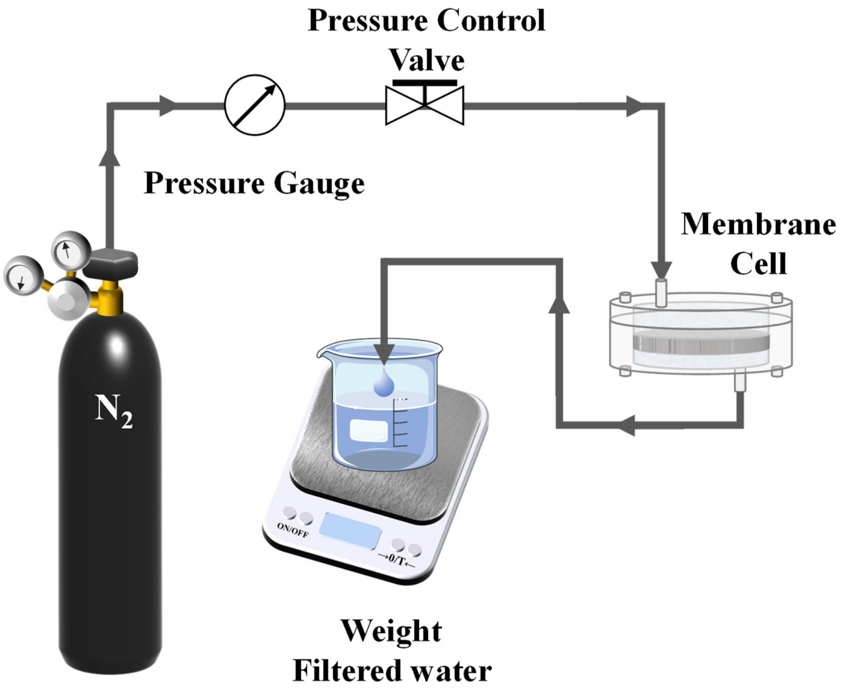

Figure S2. Schematic for PWF of the membrane measured.

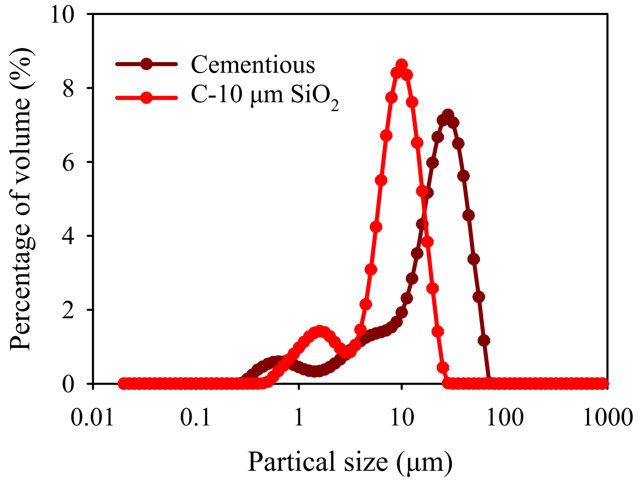

Figure S3. Particle size distribution of raw materials of the fabricated cementitious membranes fabricated.

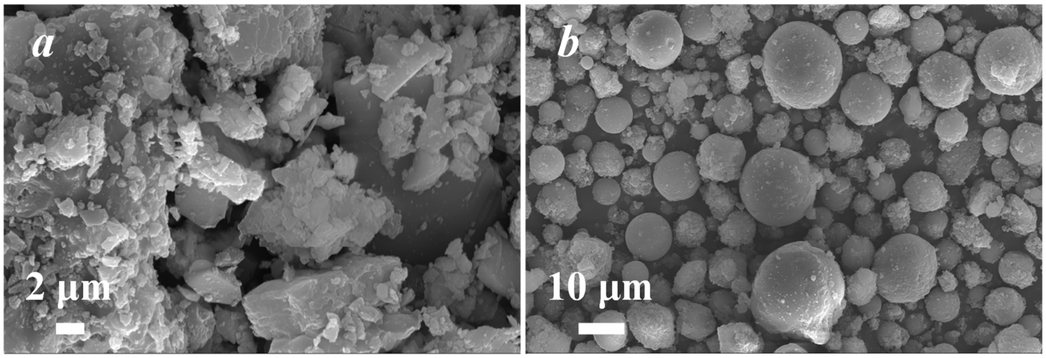

Figure S4. Apparent morphology of raw materials for membrane fabrication. (a) Cementitious powder; (b) SiO_2 particle C-10 μm.

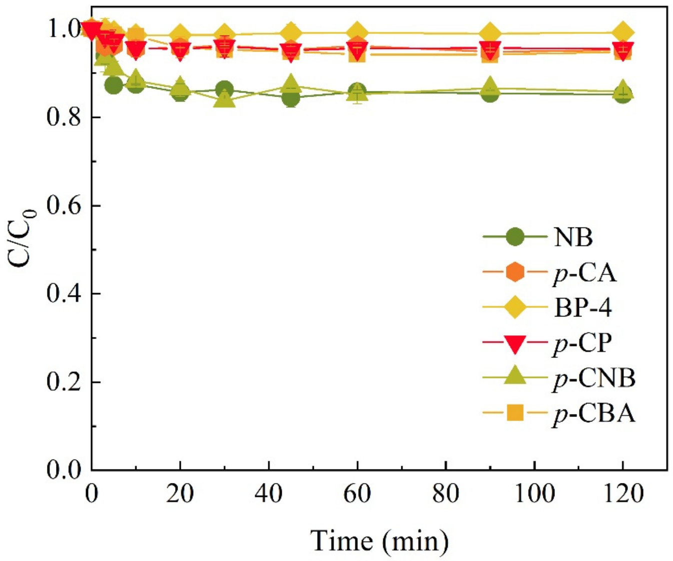

Figure S5. Adsorption of the six pollutants by the CM. Conditions: pH = 6.9±0.1, [nitrobenzene]$_0$ = [*p*-CA]$_0$ = [BP-4]$_0$ = [*p*-CP]$_0$ = [*p*-CNB]$_0$ = [*p*-CBA]$_0$ = 10 mg L^{-1}.

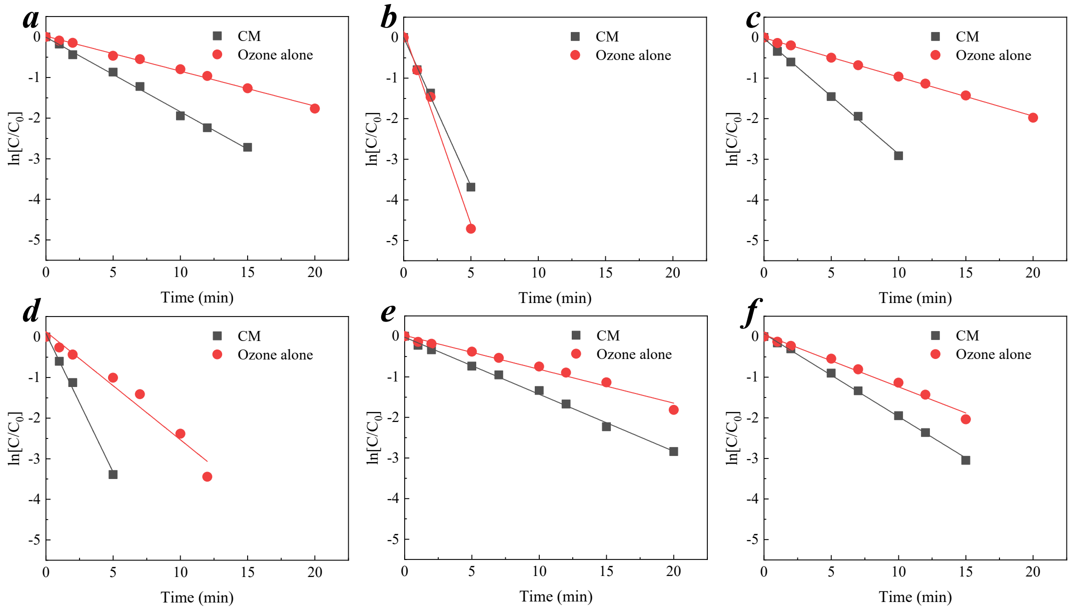

Figure S6. Reaction kinetics of organic compound ozonation catalyzed by CMs. (a) nitrobenzene; (b) *p*-CA; (c) BP-4; (d) *p*-CP; (e) *p*-CNB; (f) *p*-CBA. Conditions: pH = 6.9±0.1, [O_3] = 0.5 mg L^{-1}, [nitrobenzene]$_0$ = [*p*-CA]$_0$ = [BP-4]$_0$ = [*p*-CP]$_0$ = [*p*-CNB]$_0$ = [*p*-CBA]$_0$ = 0.064 mM.

References:

1. Kim, K. S.; Sajjad, M.; Lee, J.; Park, J.; Jun, T., Variation of extracellular polymeric substances (EPS) and specific resistance to filtration in sludge granulation process to the change of influent organic loading rate. *Desalination and Water Treatment* **2014,** *52*, (22-24), 4376-4387.
2. Sedmak, J. J.; Grossberg, S. E., A rapid, sensitive, and versatile assay for protein using Coomassie brilliant blue G250. *Analytical biochemistry* **1977,** *79*, (1-2), 544-52.
